# A comprehensive review on endocrine toxicity of gaseous components and particulate matter in smog

**DOI:** 10.3389/fendo.2024.1294205

**Published:** 2024-01-30

**Authors:** Ammara Saleem, Tanzeela Awan, Muhammad Furqan Akhtar

**Affiliations:** ^1^ Department of Pharmacology, Faculty of Pharmaceutical Sciences, Government College University Faisalabad, Faisalabad, Pakistan; ^2^ Department of Pharmacy, The Women University Multan, Multan, Pakistan; ^3^ Riphah Institute of Pharmaceutical Sciences, Riphah International University, Lahore, Pakistan

**Keywords:** smog, endocrine disruption, ambient air pollution, ozone, infertility, metabolic diseases

## Abstract

Smog is a form of extreme air pollution which comprises of gases such as ozone, sulfur dioxide, nitrogen and carbon oxides, and solid particles including particulate matter (PM_2.5_ and PM_10_). Different types of smog include acidic, photochemical, and Polish. Smog and its constituents are hazardaous to human, animals, and plants. Smog leads to plethora of morbidities such as cancer, endocrine disruption, and respiratory and cardiovascular disorders. Smog components alter the activity of various hormones including thyroid, pituitary, gonads and adrenal hormones by altering regulatory genes, oxidation status and the hypothalamus-pituitary axis. Furthermore, these toxicants are responsible for the development of metabolic disorders, teratogenicity, insulin resistance, infertility, and carcinogenicity of endocrine glands. Avoiding fossil fuel, using renewable sources of energy, and limiting gaseous discharge from industries can be helpful to avoid endocrine disruption and other toxicities of smog. This review focuses on the toxic implications of smog and its constituents on endocrine system, their toxicodynamics and preventive measures to avoid hazardous health effects.

## 1 Introduction

**Figure 1:**
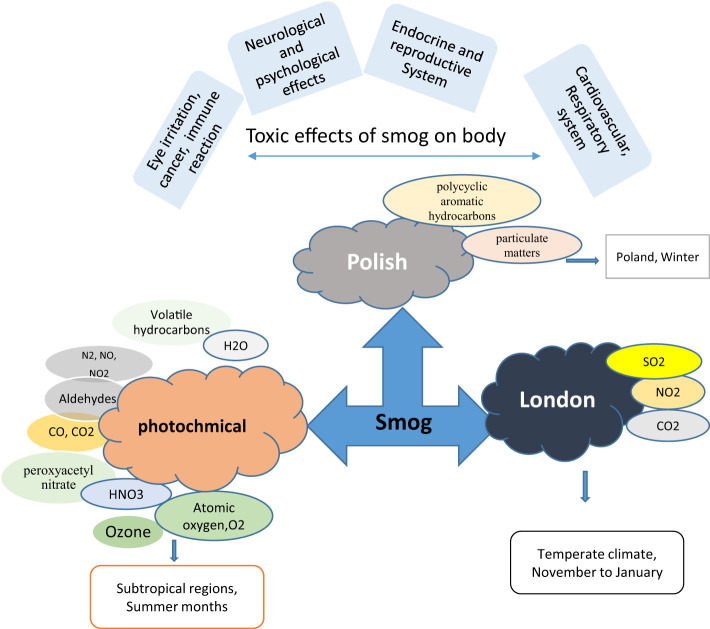
Smog types, composition, and toxic effects of smog on the body.

The term “smog” was first devised during the early 20^th^ century to define the low-level pollution covering the city of London. The word “smog” originated from two English words “smoke and fog” (1). Acidic (London smog), photochemical (Los Angeles type), and Polish smog are the three forms of smog. Acidic smog usually ascends from November to January when the atmospheric pressure is low, and air temperature remains a few degrees centigrade beyond zero due to which the concentration of pollutants increases near the ground. A combination of high humidity, low temperature, and pollutants created by the combustion of fossil fuels such as oil, gas, and coal, leads to the formation of smog (2, 3). The core components of acidic smog are oxides of sulfur, nitrogen, and carbon, carbon black, and suspended particulate matter (PM) which are produced by small heating devices when the combustion process is away from ideal conditions (**Figure 1**) (1).

Photochemical smog is principally a brown haze formed during summer due to intense sunlight and high temperature (>28-30°C) in the subtropical region (4). Biogenic and anthropogenic sources mainly contribute to photochemical smog. Biogenic sources include the production of nitrogen oxides by lightning, bushfires, microbial processes, and the vapors of volatile organic compounds produced from naturally occurring compounds such as terpenes. Nitrogen oxides formed by motor vehicles or power stations through inadequate combustion or burning of fossil fuels and volatile organic compounds are anthropogenic sources of photochemical smog. The primary constituents of photochemical smog are the oxides of nitrogen, carbon monoxide, carbon dioxide, volatile hydrocarbons, and ozone (**Figure 1**).

**Figure 2:**
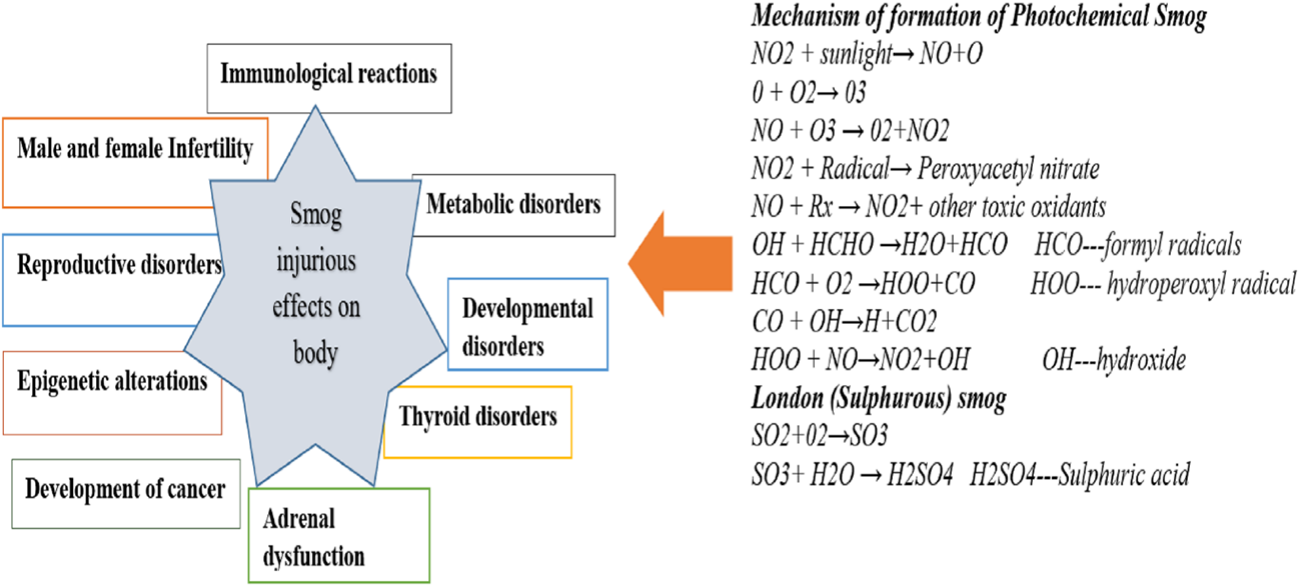
Mechanism of formation of smog and their adverse impact on the Endocrine system.

Polish smog mostly ensues during frosty season and time of eastern circulation at high pressure and weak winds (5, 6). Polish smog contains suspended PMs such as PM 1 μm, PM 2.5 μm, PM 2.5-10 μm, and various polycyclic aromatic hydrocarbons (PAH) including benzo[a]pyrene as mentioned in **Figure 2** and **Table 1**. These PMs are the most harmful components of smog the chemical composition of which varies significantly (Veras et al., 2010). PM_2.5_ is composed of sulfates, ammonia, organic compounds, elemental carbon, and metals. PM_2.5-10_ comprises of crystalline materials such as silicon, iron, calcium, aluminum, and their oxides, large salt particles, and plant debris in the atmosphere (1). The concentration of different components of smog has been summarized in **Table 1**. The mechanism of formation of different types of smog and their adverse impact on the endocrine and other systems of the human body are described in **Figure 2**.

**Table 1:** Composition and concentration of different smog components in the air.

Components of smog	Smog type	Concentration in the air during smog	Reference
Ozone (Ground level)	Photochemical smog Acidic smog	Ozone (0.8 ppm) Ozone (0.4 - 0.8 ppm) Ozone (0.8 -1.0 ppm)	(27) (7) (8)
Particulate matter (Surface and volume Concentration) a complex mixture of solid particles and liquid Particulate with a diameter ≤2.5 μm (PM_2.5_)	Acid smog Polsih smog Photochemical smog	Approx. 949649206 μm^2^/cm^3^ PM_2.5_ 4.90 to 38.07 ppm PM_10_ 7.61 to 38.49 ppm PM_10_ 10 µg/m^3^ in Asian cities	(28) (29)
Nitrogen dioxide	Photochemical smog	Approx. 200 ppb	UCAR Center of science education
Benzopyrene	Acidic smog	Approx.61.6 ng/m^3^ Approx. 3.64 ng/m^3^	(9)
Carbon monoxide	Photochemical smog Polish smog	Approx.160 ppm	(29)
Carbon black	Acidic smog Polish smog	More than 20 μg/m3	(10)
Sulphur dioxide	Polish smog Acid smog Photochemical smog	Approx. 0.75 ppm	(11)
Peroxyacetyl Nitrate (PAN)	Acid smog	Approx. 37 ppb	(29)
Nitric acid	Polish smog	Approx. 49 ppb	(29)
Formic acid	Acidic smog	Approx. 19 ppb	(28)
Formaldehyde	Polish smog	Approx. 71 ppb	(28)
Phthalate diesters	Acidic smog	10-100 ng/m^-3^	(30) (12)
Alkylphenols	Photochemical smog	Approx.1 ng m^-3^	(12)
Polychlorinated biphenyls (PCBs)	Acidic smog	Approx. 0.1ng m^-3^	(12)
Biphenol C_12_H_10_	Photochemical smog Acid smog	Approx. 0.1ng m^-3^	(12)

**Figure 3:**
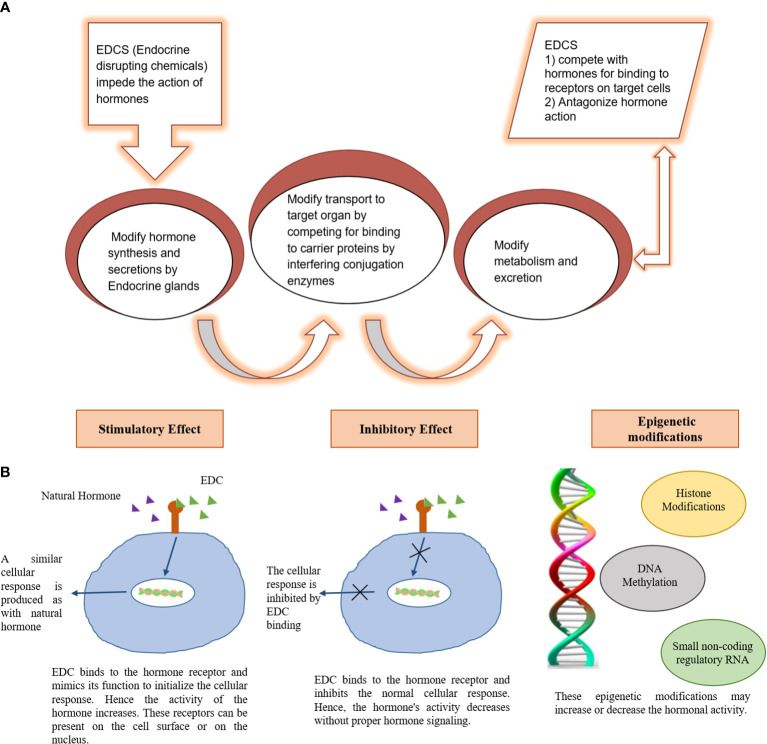
Mechanism of endocrine damage by Endocrine disrupting chemicals (EDC). **(A)** Mechanism of EDC induced endocrine disruption **(B)** molecular basis for EDC induced endocrine disruption.

The developing countries of South Asia, Southeast Asia, North Africa, Middle East, and South America are mostly affected by air pollution which display low air quality due to widespread industrial activities, motor vehicles, and fossil fuel burning (13). An increase in air pollution has reduced the quality of life, caused air-borne diseases, and reduced the life expectancy (14). In addition, gaeous components and PM_2.5_ present in smog are associated with endocrine disruption in affected individuals. Endocrine-disrupting chemicals (EDCs) in smog are frequently linked to the reduction in sperm quality, irregular menstrual cycles, and fertility issues. EDCs also interfere with the functions of thyroid and adrenal glands (15) (16). EDCs alter the synthesis and transport of endocrine hormones by affecting the conjugating enzymes or competing for binding to carrier proteins (17). Furthermore, EDCs alter the metabolism of hormones and compete for binding sites by mimicking steroid hormones, especially estrogens and androgens (Darbre 2018). The mechanism of EDCs-induced endocrine disruption is depicted in **Figure 3**. Therefore, the current review is aimed at identifying the toxic implications and toxicodynamics of smog and its constituents related to the endocrine system. Moreover, this review appraises the available data regarding adverse health implications on humans including the special population groups such as the elderly, young adolescents, and pregnant individuals.

## 2 Methods

**Figure 4:**
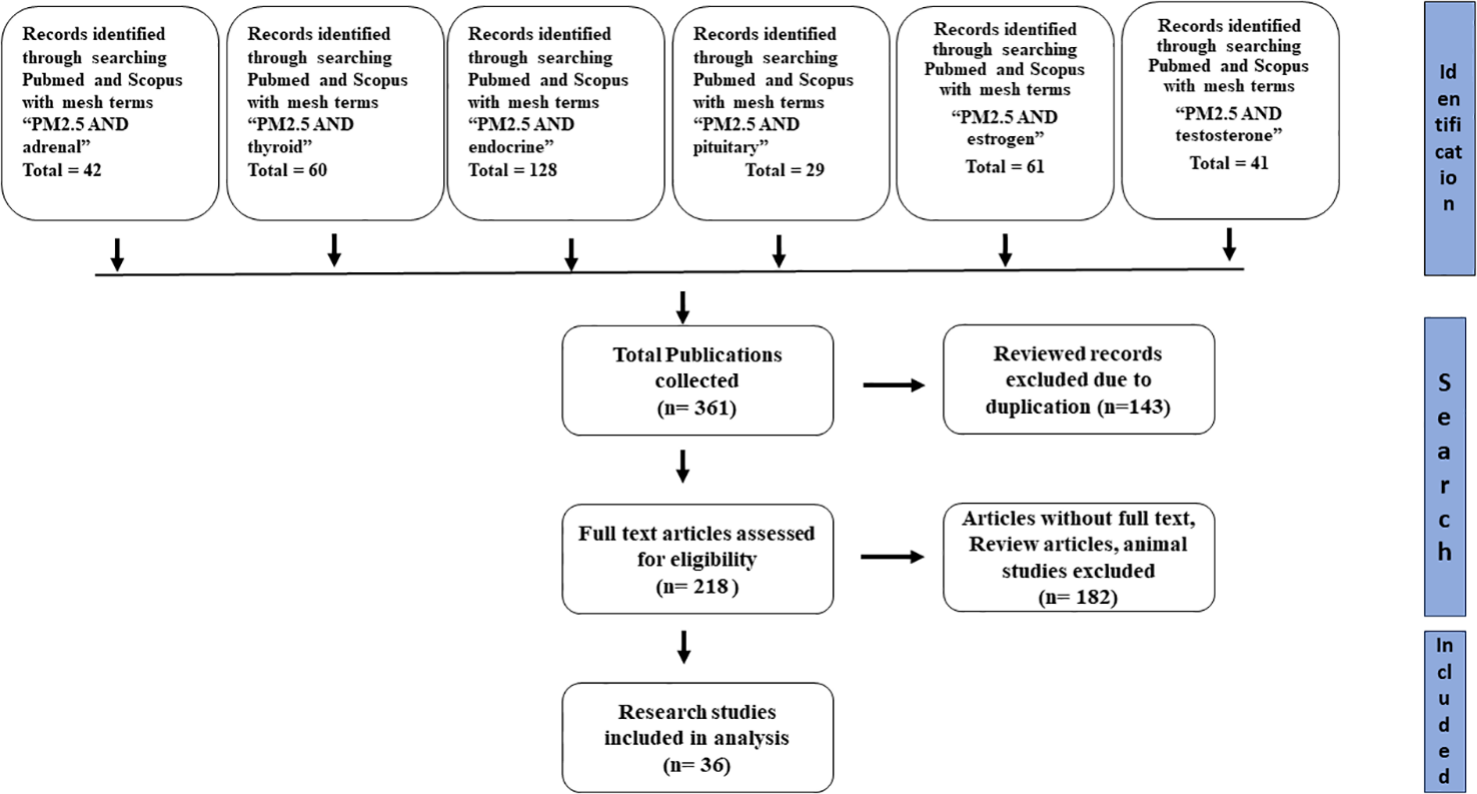
Prisma diagram for selection, review and analysis of studies.

For finding relevant studies regarding the endocrine disruptive effect of smog and its constituents, a wide range of search terms, including but not limited to “ smog”, “smog constituents”, “Acidic smog”, Polish smog” and “Photochemical smog” were searched from different databases such as Scopus, google scholar, and PubMed. For the study of the effects of PM_2.5_ on various endocrine systems, the search terms such as “PM_2.5_ and endocrine”, “PM_2.5_ and pituitary”, “PM_2.5_ and adrenal”, “PM_2.5_ and thyroid”, “PM_2.5_ and estrogen”, “PM_2.5_ and testosterone” were used to extract data from Pubmed and Scopus. After identifying all records, duplicate records, review articles and animal studies were excluded. Moreover, those articles with missing full text were also excluded from the study. The remaining articles were assessed for the study participants, exposure type, and results. The flow diagram depicting the process for the selection of research studies is shown in **Figure 4**.

## 3 Endocrine disruptive effects of gaseous constituents of smog

Smog has extensively affected the quality of life by not only instituting numerous health issues but also aggravating the already existing diseases. Gaseous components of smog include ozone, carbon monoxide (CO_2_), sulfur dioxide, nitrogen oxides, and others.

### 3.1 Endocrine disruptive effects of ozone

Several environmental toxicants produce acute and stress-related changes through direct or indirect action (18). Several gases such as ozone and sulfur dioxide alter the metabolism of carbohydrates. Acute ozone exposure causes release of stress hormones through the suppression of hypothalamic-pituitary axis (HPA) (19).

#### 3.1.1 Ozone and stress hormones

Long-term elevation of stress hormones i.e., leptin and corticosteroids in the circulation results in metabolic diseases and systemic inflammation (20). The HPA axis is involved in acute O_3_-induced extra-pulmonary effects. In addition to the induction of glucose intolerance, O_3_ increases the level of leptin and epinephrine (21). Leptin and epinephrine play a pivotal role in the regulation of body temperature and weight (22). Stress hormones target the liver and pancreas to alter glucose and lipid metabolism through the activation of cellular glucocorticoid and adrenergic receptors (23, 24).

#### 3.1.2 Dysfunction of parathyroid gland

The exposure of 0.75 ppm O_3_ altered the function of parathyroid glands (25). The 48 hours of exposure to 0.75 ppm O_3_ in the rabbits’ parathyroid glands resulted in hyperplastic parathyroiditis. Inhalation of O_3_ initiated the autoimmune reaction that resulted in the permanent destruction of the parathyroid glands. At the initial exposure, parathyroid glands were compact and in a cluster arrangement but at a later stage, they were congested and enlarged due to ozone exposure (26). However, evidences of ozone-induced parathyroid toxicity in human are unavailable.

#### 3.1.3 Reproductive toxicity of ozone in males

Ozone also induces plasma membrane remolding by stimulating the adrenergic nervous system through the release of catecholamine (20). LH is also responsible for the production of testosterone from the Leydig cell in males.

#### 3.1.4 Reproductive toxicity of ozone in females

Ozone exposure reduces progesterone and increases estrogen by post-exposure effect on pituitary gland to alter luteinizing hormone (LH) (31). Exposure to 0.3 ppm O_3_ causes menstrual cycle disturbance in females by fluctuating LH release. These disturbances affect ovulation, fertilization, maintenance of endometrial lining, and implantation of fertilized ovum. Exposure to O_3_ at level more than 0.3 ppm caused menstrual disturbances that led to a sterile cycle. In addition, O_3_ exposure is inversely related to the ovarian reserve. It is found that exposure to O_3_ is positively associated with low excretion of anti-Muellerian hormone (AMH), an important marker for ovarian reserve (32). However, some studies showed that exposure to O_3_ was effective in treating female infertility. It can protect from inflammatory problems such as endometritis, and vaginitis, and reduces the chances of ischemia-induced injury in ovaries (33). Another study has shown that O_3_ therapy might enhance ovarian function by improving oocyte quality and altering the genes involved in the synthesis of steroidal hormones (34). However, ozone, when used as a therapeutic agent, should be generated in controlled concentration from pure oxygen and the intake should be monitored. Despite the compelling therapeutic evidence, future research is necessary to critically explore whether the effects of O_3_ in the female reproductive system are beneficial or not (35).

#### 3.1.5 Developmental toxicities of ozone

**Figure 5:**
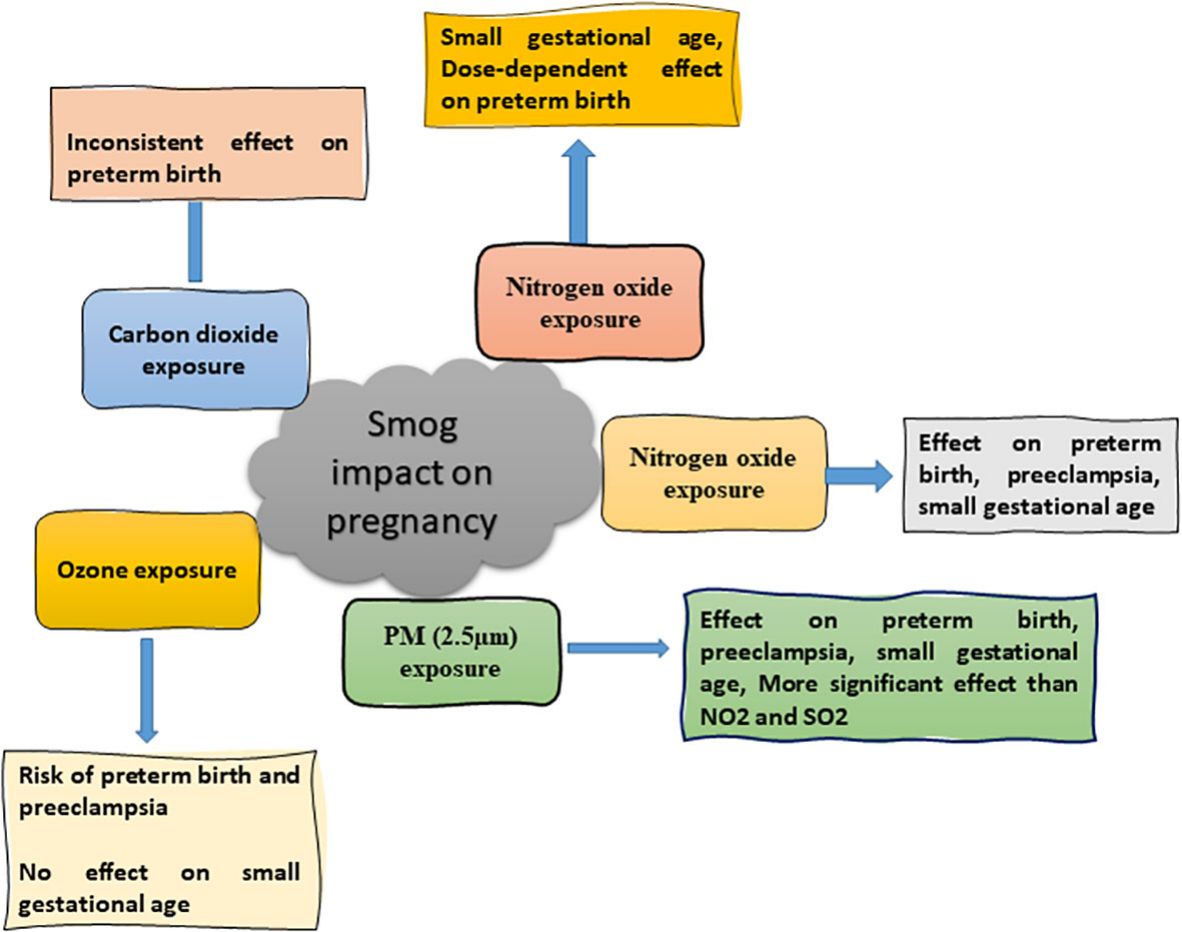
Effect of smog exposure during pregnancy.

The gaseous constituents of smog have significant adverse consequences on pregnancy if a pregnant mother is exposed to outer ambient air pollutants for a long duration due to changes in hormones’ level (36). Preterm birth, preeclampsia, and small-for-gestational age (SGA) are the adverse outcomes of exposure to smog air pollutants (**Figure 5**). SGA is described as infants with a weight less than 10% at a specified gestational age (37). Previous study showed that the incidence of preterm birth and pre-eclampsia were 4.4 and 2.7% respectively in pregnant women upon long term exposure to smog air, especially ozone. It was further confirmed that a positive association was found between the first trimester, O_3_, and preterm birth. A positive correlation was also evident between O_3_ and pre-eclampsia when the concentration of O_3_ was increased to more than 10 µg/m^3^ (38).

#### 3.16 Association of ozone with insulin resistance

Air-borne pollutants are highly associated with the risk to diabetes. Acute exposure to O_3_ changed the metabolism of rats and increased the risk factors associated with metabolic alterations. Additionally, it was found that a high O_3_ level altered glucose homeostasis by changes in the insulin signaling pathway and liver endoplasmic reticulum stress in rats (39).

### 3.2 Endocrine disruptive effects of sulfur dioxide

**Figure 6:**
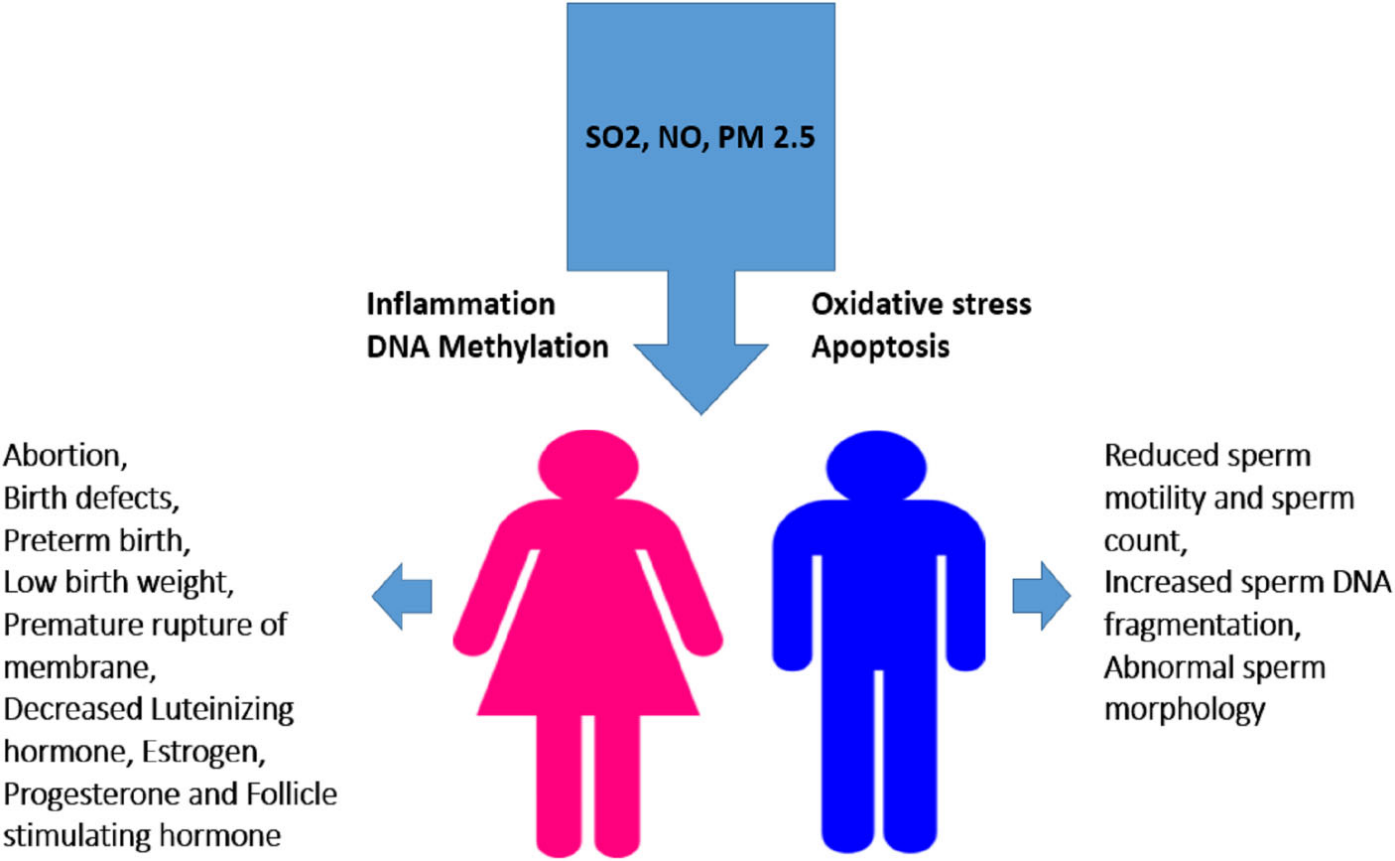
Effect of smog exposure on the reproductive system in males and females. Exposure to SO2, PM2.5 or NO triggers various cell-damaging processes such as oxidative stress, inflammation, apoptosis, and DNA methylation leading to altered sperm quality and concentration in males while induce abortion and alters hormone level in females. Low birth weight, birth defects and hypertension in mothers are the consequences of their exposure during pregnancy etc.

Exposure to Sulfur dioxide during pregnancy can cause birth defects and abortion. Short-term exposure a high concentration of SO_2_ is life-threatening (40). Its exposure to human causes reproductive and developmental effects as mentioned in **Figure 6**. A study carried out in Finland’s industrial areas revealed that SO_2_ exposure had resulted in spontaneous abortion (41). Another study in China demonstrated a link between exposure to SO_2_ during pregnancy and reduced infants’ birth weight (42). Exposure of pregnant rabbits to SO_2_ resulted in minor skeletal variation and delayed bone hardening (43). In addition, exposure to SO_2_ caused a significant, dose-related decrease in plasma insulin levels (44).

Sulfur dioxide exhibits reproductive toxicity in male animals. An investigation in the Czech Republic showed that a high-level exposure to SO_2_ caused sperm abnormalities such as a decreased ability to move (45). In an experimental study in mice, it was found that an exposure to 5 mg/m^3^ SO_2_ increased sperm malformation, decreased sperm count, and exhibited aberrant pathological changes in testicles. Additionally, mice exposed to SO_2_ also increased TUNEL-positive cells, caspase-3 activity, spermatogenic cell counts, hydrogen peroxide (H_2_O_2_) and melondialdehyde (MDA) content, and decreased superoxide dismutase (SOD) activity. It was demonstrated that exposure to SO_2_ altered the expression of steroidogenic-related genes (LHR, StAR, and ABP), lowered serum testosterone levels, and altered the mRNA levels of Bax and Bcl-2 in mice. In conclusion, exposure to SO_2_ exhibited male reproductive toxicity through induction of apoptosis, lipid dysregulation, and generation of reactive oxygen species (ROS). These outcomes provided a fresh theoretical framework for understanding the interference of SO_2_ with spermatogenesis and infertility (46).

### 3.3 Endocrine disruptive effects of carbon monoxide and nitrogen oxides

Gaseous neuromodulators, such as nitrogen monoxide (NO) and carbon monoxide (CO), regulate the hypothalamic release of neuropeptides. The stimulatory and inhibitory effects on the HPA depend upon diverse factors, such as type of stress, intensity of stress, and species. NO regulates the non-adrenergic and non-cholinergic relaxation of smooth muscles in the corpora cavernosa and gastrointestinal tract (47). Exposure to CO inhibits the secretion of corticotropin-releasing hormone (CRH) and oxytocin while raising the secretion of gonadotrophin-releasing hormone (GnRH) and prostaglandin (PGE2) in the rat hypothalamus. Stimulation of the HPA axis increases the body temperature which is termed stress fever (48).

#### 3.3.1 Reproductive toxicity of NO_2_ in males

NO_2_ is one of pivotal factors that contribute to male infertility. Boggia and his coworkers reported that NO_2_ exposure had led to reduced sperm motility, quality and quantity in exposed males as compared to non-exposed males. Negative effects on sperm quality were noticed even if NO_2_ concentration was below than recommended allowed limit (49, 50). Investigation on the male genome has shown that NO_2_ had broken the strands of the sperm DNA ultimately leading to infertility (51).

#### 3.3.2 Carcinogenicity of NO_2_ in females

Outdoor exposure to NO_2_ is closely associated with lung cancer in females through increased activation of estrogen receptors. A few studies have demonstrated that estrogen receptor activation promoted tumor formation by several genomic and non-genomic pathways (52). Female lung cancer is closely associated with the activation of estrogen receptor pathway and NO_2_ is implicated to activate this pathway (53).

#### 3.3.3 Adverse effects of NO_2_ during pregnancy

Various studies have revealed the deleterious effects of exposure to NO_2_ against pregnancy. Exposure to air pollutants has resulted in increased DNA fragmentation. These toxicants also cause alterations in the placenta’s DNA. Early pregnancy exposure to NO_x_ changed the placental DNA methylation leading to placental immaturity (54, 55). Chronic interaction with NO_x_ is also linked with diabetes mellitus. Leiser et al. reported that exposure to NO_2_ up to 100 ppb for 7 days had increased the chances of miscarriage by up to 16% (56).

## 4 Endocrine disrupting effects of particulate matter (PM_2.5_) in smog

The PMs are comprised of dust, soil, acids, metals, and organic compounds. The PMs are categorized according to their size or diameter i.e., PMs of 2.5-10 µm are considered coarse particles, PMs of less than 2.5 µm are fine particles, and PMs of less than 0.1 µm are ultrafine particles (57). A high concentration of PMs, usually PM_2.5_ dust, constitutes smog in the winter season. PMs produce systemic effects by influencing metabolic homeostasis. Long-term exposure to PM increases the disease progression (58, 59). Exposure to PM_2.5_ induces insulin resistance and stimulates the HPA axis which consequently intensifies glucocorticoid production. In addition, PM_2.5_ increases response of the HPA axis to psycho-social stress especially in adolescent girls who experience high social stress (60).

### 4.1 PM_2.5_ and thyroid dysfunction

Thyroid hormones such as thyroxine (T_4_), and triiodothyronine (T_3_) are secreted by the thyroid gland under the influence of thyroid stimulating hormone (TSH) secreted by pituitary glands. Industrial chemicals, tobacco smoke, and air pollution impact thyroid hormone production. Thyroid hormones are responsible for fetal growth, metabolism, and neuro-development. Low and high levels of thyroid hormone unfavorably affect child’s growth. PM_2.5_ exposure may change the thyroid function of a newborn (20, 61, 62). It was found that the exposure to PM_2.5_ ≥ 16 µg/m^3^ concentration in air had raised the T_4_ level to 7.5 percent in the blood. Moreover, PM_2.5_ increased the conversion of T4 to T3 (20, 63). This might be due to the changes in genes encoding for steroid hormone biosynthesis and glycerolipid metabolism caused by PM_2.5_ (64).

Exposure to pollutants at the time of birth or during childhood may increase the risk of cancers in children, the most common are leukemias and lymphomas. Ambient PM air pollution is also related to a higher incidence of thyroid cancer (65). Moreover, other studies have revealed that the chronic exposure to air pollution may produce alterations in normal ovarian function e.g., estrogen-like effect or gene mutation, which can lead to ovarian cancer (66). Additionally, transplacental exposure to PM is connected to higher placental mutation rate and such epigenetic alterations may be a reason for placental carcinogenicity (55).

### 4.2 PM_2.5_ associated insulin and glucocorticoid resistance

The toxic effect of ambient air pollution may lead to insulin and glucocorticoid resistance by interfering with the signaling pathways involved in inflammation. It was found that increased level of TNF-α caused inhibition of the insulin signaling pathway (67). PM_2.5_-mediated insulin resistance results in the accumulation of fatty acids in the liver and dysregulates glucose utilization by skeletal muscles through increased expression of C-C Chemokine receptor (CCR-2) and reduced GLUT-4 (68). Another study suggested that the blockage of the glucocorticoid or HPA axis by exposure to smog pollutants may lead to the overproduction of cytokines and inhibition of the glucocorticoid-regulated gene, CYP3A5 (69).

### 4.3 Reproductive toxicity of PM_2.5_ in males

PM_2.5_ exposure can lead to reproductive damage in males by inducing apoptosis and inflammatory pathways. PM_2.5_ adversely affects the male reproductive function (Liao et al., 2019). Exposure to PM may result in increased expression of various cytokines and methylation at CpG sites. Exposure to PM_2.5_ specifically increased the expression of MCP-1, MCP-3, CD40, FGF-2, and other related genes in young adults (70). Moreover, interleukins, Toll-like receptor genes, and genes related to apoptosis are upregulated due to smog exposure. Expression of xenobiotic genes and cytochrome P450 genes is altered by exposure to PM_2.5_. Genes associated with cancer development such as TGFβ are overexpressed resulting in the activation of the linked signaling pathways (71).

### 4.4 Reproductive toxicity of PM_2.5_ in females

PM_2.5_ damages ovarian granulosa cells and oocytes by decreasing the levels of AMH and increasing the expression of inflammatory and apoptotic proteins (72). Smog pollutants can delay normal conception and *in-vitro* fertilization (IVF) (73). The effects of PM_2.5_ on the female reproductive system include the dysfunction of ovaries and transportation to the embryo or mutation in the embryo’s DNA (74). Data from an infertility clinic showed that exposure to PM_2.5_ was associated with loss in normal ovarian function resulting in infertility (75).

### 4.5 Effect of PM_2.5_ on pregnancy and fetus development

Congenital hypothyroidism (CH) is the most common endocrine disorder in newborns, affecting 1 in 2000-4000 newborns. Air pollution is also responsible for CH (76). It was found that a exposure to a high concentration of PM is associated with CH which causes delayed physical and mental development and may affect the normal functioning of kidneys, lungs, and heart. The risk of preterm birth, fetal death, low birth weight, congenital imperfections, and macrosomia fetus is increased due to PM_2.5_ and PM_10_ exposure. Moreover, the reduction in exposure to PM_2.5_ was associated with an increased survival rate of newborns (**Figure 5**) (38). Similarly, an exposure to PM_2.5_ up to 10 mg/m^3^ for seven days was accompanied by miscarriage (56). The studies regarding the effect of smog and its constituents on different endocrine systems are listed in **Table 2**.

**Table 2:** The effect of PM_2.5_ exposure on endocrine systems of the male and female individuals.

Serial No.	Exposure type	Study participants	Toxic effects	Reference
1.	PM_2.5_ at 3.7 μg/m^3^	Prenatal exposure	increased neonatal TSH levels	(77)
2.	Ambient PM_2.5_ at 8.13 µg/m^3^ concentration	Salivary cortisol output during pregnancy in a	decline in cortisol throughout the day with increasing exposure	(78)
3.	NO_2_ at 24.4 ± 14.0 ppb and PM_2.5_ at 55.6 ± 41.5 μg/m^3^/day for 1-14 days	COPD patients to the neuroendocrine response in COPD patients.	Increase in CRH, ACTH, and norepinephrine, and decreases in cortisol and epinephrine	(79)
4.	PM_2.5_	Prenatal exposure during third trimester of pregnancy	Increased depression risk and induces activation of the HPA axis	(80)
5.	PM_2.5_	Young adolescent girls	heightened HPA-axis stress responsivity, Increased biological sensitivity to social stress	(60)
6.	PM_2.5_	Exposure during pregnancy	Dose dependent increase in cortisol levels in cord blood, as the distance of exposure increased, the decrease in cord-blood cortisol level	(81)
7.	10 ppb of NO_2_, PM_2.5_	45-85 years old participants	9.7% higher wake-up cortisol associated with a 10 ppb NO_2_, the cortisol curve became flatter over 5 years.	(82)
8.	PM_2.5_ at 41.1 μg/m^3^	Young adults	NO_3_ ion was still significantly associated with CRH, Increased CRH, ACTH and cortisol.	(83)
9.	PM_2.5_	Pregnant individuals	first-trimester exposures were associated with mild thyroid dysfunction throughout pregnancy, dose dependent increase in toxicity	(84)
10.	PM_2.5_	Elderly women with mean age of 73.5 ± 3.0 years	Higher risk of dementia in women with three estrogen receptors with SNPs	(85)
11.	PM_2.5_, O_3_ and NO_2_	Air pollutants and hormone-assessed pubertal development	No statistical effect on hormone levels of E2 and testosterone	(86)
12.	Three-years exposure to PM_2.5_	Dementia-free women aged 80 and older	episodic memory declines mediated by depressive symptoms	(87)
13.	PM_2.5_	Black women	not associated with a higher risk of breast cancer except for some geographic areas	(88)
14.	PM_2.5_	Pre-conception and early prenatal periods	can lead to altered steroid adaptation during the state of pregnancy	(89)
15.	PM_2.5_, NO_2_	Women with 1-year familial breast cancer risk	Increased risk among women with a higher familial risk with NO_2_ only	(52)
16.	Improved air quality with PM_2.5_	Exposure for 3 years in older women of less or more than 80 years with no dementia	improvement in long-term AQ in late life was associated with slower cognitive declines in older women	(90)
17.	NO_2_, CO, SO_2_, or PM_2.5_, PM_10_	Female adults aged ≥ 40 years	Increased risk of osteoporosis in female with PM_10_ only	(91)
18.	PM_2.5_	All cause ovarian cancer patients 18–79 years	PM2.5 concentrations were associated with an increased risk of all-cause mortality.	(92)
19.	PM_2.5_ 4.9 to 17.5 µg/m3	31 years old female participants	Weak inverse associations with POM, no dose response relationships	(93)
20.	PM_2.5_ and PM_10_ through road exposure	150 mother-newborn pairs	Directly related to increased cortisol levels in cord-blood	(81)
21.	NO_2_ at 10 ppb and PM_2.5_	45–85 years old participants	Higher wake-up salivary cortisol with NO_2_ only which flattened over 5 years	(82)
22.	PM_2.5_ in residential areas	Women in third trimester of pregnancy	More severe depressive symptoms and activation of HPA axis	(80)
23.	NO_2_, O_3_ and PM_2.5_	Obese Latino children and adolescents	Higher O_3_ exposure caused higher morning cortisol PM_2.5_ exposure (4–10 months) caused lower serum morning cortisol.	(94)
24.	PM_2.5_ at 41.1 μg/m^3^	CRH, ACTH and cortisol in young adults	Water-soluble inorganic constituents especially, NO_3,_ caused stronger activation of HPA axis	(83)
25.	PM_2.5_, PM_10_	Participants from couples who underwent *in-vitro* fertilization treatment	PM_2.5_ increased seminal testosterone and malondialdehyde, and reduced sperm progressive motility.	(95)
26.	PM_2.5_	Prenatal exposure to pregnant individuals	Reduced anogenital distance of new born	(96)
27.	PM_2.5_	Pregnant individuals in third trimester	Increased in cord blood levels of 17α-hydroxy-pregnenolone	(97)
28.	PM_2.5_, SO_2_ and CO	women undergoing assisted reproductive procedure	Reduced testosterone, progesterone and FSH	(98)
29.	PM_2.5_, NO_2_, SO_2_, CO, and O_3_	Effect on testosterone, FSH, LH, E2, PRL in men aged 20–55 years	immediate and short-term cumulative PM2.5 reduced testosterone.	(99)
30.	PM_2.5_, NO_2_ and PM_10_	infertile men	PM_2.5_, and NO_2_ were negatively associated with sperm morphology.	(100)
31.	PM_1_, PM_2.5_, and PM_10_	Rural adult male and female	PM_2.5_ increased the testosterone in male and reduced progesterone in both male and female.	(101)
32.	PM_2.5_ particles and bound eight PAHs	Male college students	LMW-PAHs negatively affected sperm morphology, PAHs increased sperm motility.	(102)
33.	single-day and cumulative effects of air pollutants of PM_2.5,_ SO_2_, and NO_2_	Male young adults	PM_2.5_ concentrations were positively associated with E2. SO_2_ and O_3_ reduced E2.	(103)
34.	PM_2.5_, CO, NO_2_, PM_10_	Infertile men	PM_2.5_, CO and NO_2_ were negatively associated with the level of testosterone, PM_2.5_ also caused immature chromatin	(104)
35.	PM_2.5_	fertile men of 20-45 years	Decreased sperm motility, total motility, and sperm quality	(105)
36.	PM_2.5_, PM_10_, SO_2_, NO_2_, CO, and O_3_ 14-18 µg/m^3^ During the third trimester	Women with preterm birth information or low-birth weight	Low birth weight risk was associated with PM_2.5,_ NO_2_, and O_3_	(42)

ACTH, adrenocorticotropic hormone; CRH, Increased corticotropin releasing hormone; COPD, Chronic obstructive pulmonary disease; E2, Estradiol; FSH, follicle stimulating hormone; HPA, Hypothalamus pituitary axis; LH, luteinizing hormone; LMW, low molecular weight; PAH, Poly aromatic amines; POM, Polycystic ovarian morphology; PRL, prolactin; SNP, Single nucleotide polymorphism.

### 4.6 Effect of PM_2.5_ on fertility

Various EDCs present in the environment exert a negative effect on male reproductive health. These factors interfere with the normal hormonal balance especially a reduction in semen production (106). A recent study determined that PM_2.5_ exposure changed the integrity of the blood-testis barrier by ROS production and caused the loosening of tight junctions (107). The PAHs and heavy metal ions present in the particulate matter exert estrogenic, antiestrogenic, and antiandrogenic activities to disrupt normal hormone functions (59).

### 4.7 Obesogenic potential of PM_2.5_

During pregnancy and lactation, PM_2.5_ exposure is linked to metabolic disorders in neonates. Exposure to PM_2.5_ can elevate the blood pressure in the offspring which is mediated by alterations in the transcriptional activity, DNA methylation, oxidative stress and inflammatory response (108). Studies have shown that maternal exposure to PM may increase the incidence of obesity and other metabolic disorders such as fatty liver disease, diabetes militus, and insulin resistance (109). Methylation of leptin promotor and increased formation of hypothalamic neuropeptide Y in males are responsible for PM-induced obesity. PM exposure causes a reduction in the birth weight of the offspring but in the long run it induces obesity during adulthood (110).

### 4.8 Association of PM_2.5_ with depression, anxiety, and memory loss

Several studies have demonstrated that the risks of depression, anxiety, and dementia are increased in adolescent girls, elderly individuals, and pregnant females exposed to PM_2.5_. It was found that the risk of depression in pregnant individuals exposed to PM_2.5_ during the third trimester was increased due to altered HPA axis (80). Moreover, pregnant individuals exposed to PM_2.5_ displayed dose-dependent mild thyroid dysfunction which also contributed to depression (84). Estrogen plays a substantial role in cognitive function. Different studies have reported that healthy elderly females were associated with a higher risk of depression and episodic memory decline while those with genetic polymorphism in estrogen receptors showed a higher risk of dementia (85, 87). In addition, adolescent females exhibited a higher response to social stress and showed symptoms of anxiety due to exposure to PM_2.5_ (60).

## 5 Polycyclic aromatic hydrocarbons in smog

PAHs, for instance, benzo[a]pyrene and dimethyl Benz[a]anthracene are the components of Polish smog that are deposited on land and water in dry form. Benzopyrene exists in food, dust, air, water, soil, and smoke. The air contaminated with benzopyrene exerts a profound adverse impact on the health of children and employees of aluminum and coke–oven factories (111). Benzopyrene belongs to the class 1 carcinogen and is a strong mutagenic, genotoxic, teratogenic, anti-fertility and neurotoxic agent. It involves in depurination of DNA, generation of ROS, stimulation of aryl hydrocarbon receptors (AhR), and numerous other epigenetic changes that collectively result in the toxic effects.

### 5.1 PHAs and thyroid dysfunction

TSH secreted by the pituitary gland activates the synthesis of T4 and T3 in the thyroid gland which are essential for regulating development, growth, morphogenesis, basal metabolism, reproduction, and osmoregulatory functions (112). Some studies have suggested that PAHs interrupt the metabolic functions of thyroid hormone (113).

Some PAH like 3-methylcholanthrene, are carcinogenic, and disruptors of thyroid function in animals which alter the structure of the thyroid gland and synthesis of thyroid hormones (114). Small thyroid follicular with cuboidal or columnar epithelial cells have additional secretory activity as compared to large follicles with squamous epithelium. Exposure to benzo[a]anthracene caused thyroid follicles to become large with short epithelium. Furthermore, exposure to benzo[a]anthracene changed the plasma levels of TSH, T4 and T3. In fish, exposure to these EDC caused enlargement of the thyroid gland with an indication of hypothyroidism (113, 115).

Likewise, polyhalogenated aromatic hydrocarbons (PHAH) are considered EDC due to alteration in the thyroid and retinol functions in birds (61, 116). Additionally, these hydrocarbons may interfere with thyroid transport proteins i.e., transthyretin (TTR) and retinol binding protein (RBP). Some PHAHs have structural similarities to T4 and have greater binding affinity to TTR than T4 (117).

### 5.2 Reproductive toxicity of PHAs

Benzopyrene contamination in the atmosphere is a leading threat to health due to hormone receptor binding and activation, post-receptor signaling pathway, and involvement of co-factors (118, 119). Benzopyrene impedes the functions of nuclear hormone receptors i.e., estrogen, androgen, progesterone, thyroid, and retinoid receptors, membrane receptors, non-steroid receptors, and orphan receptors (118, 120).

Testosterone production is stimulated by LH produced by the pituitary gland in response to GnRH (121). Benzo[a]anthracene reduced the serum and intratesticular testosterone level (122). Tian et al. reported that exposure to benzo[a]pyrene had reduced the level of 17β-estradiol and progesterone in ovaries and decreased the expression of metabolizing enzyme, hydroxysteroid dehydrogenase (123).

### 5.3 Developmental toxicity of PHAs

The production of hormones and exchange of nutrients take place through syncytiotrophoblasts (STs) that are exposed to maternal blood. These STs are formed by the fusion of cytotrophoblasts. Any abnormal change in the formation of STs may cause premature birth or abnormal fetal development. It is previously explored that the trophoblast exposure to formaldehyde was associated with oxidative stress that adversely affected the differentiation and fusion of trophoblasts (124). Cathey et al. studied 659 pregnant women and observed a positive association between PAH and cortisone-releasing hormone, progesterone, and thyroid T_3_ hormone; however, a negative relation was found with testosterone that consequently affected the physiology of pregnancy (125). Prenatal exposure to PAHs may lead to adverse effects on the fetus, including weight loss and, reduced height and head circumference at birth. PAHs have been found in pregnant women’s blood which provides evidence that they can cross the placenta (126).

## 6 Endocrine toxicity of other trace compounds in smog

Bisphenols, phthalates, and aldehydes are included in the list of EDCs that constitute the smog. These chemicals have the potential to strongly bind to the hormone receptors resulting in the blockade of the hormone function. For instance, bisphenols can bind to the estrogen receptors and reduce the production of estradiol, estrone, testosterone, and androstenedione (127). Some studies have reported a reduced level of progesterone and estradiol in animals exposed to phthalates. In these studies, GnRH was found to be elevated, indicating that these chemicals disrupt the function of the HPA (128).

**Figure 7:**
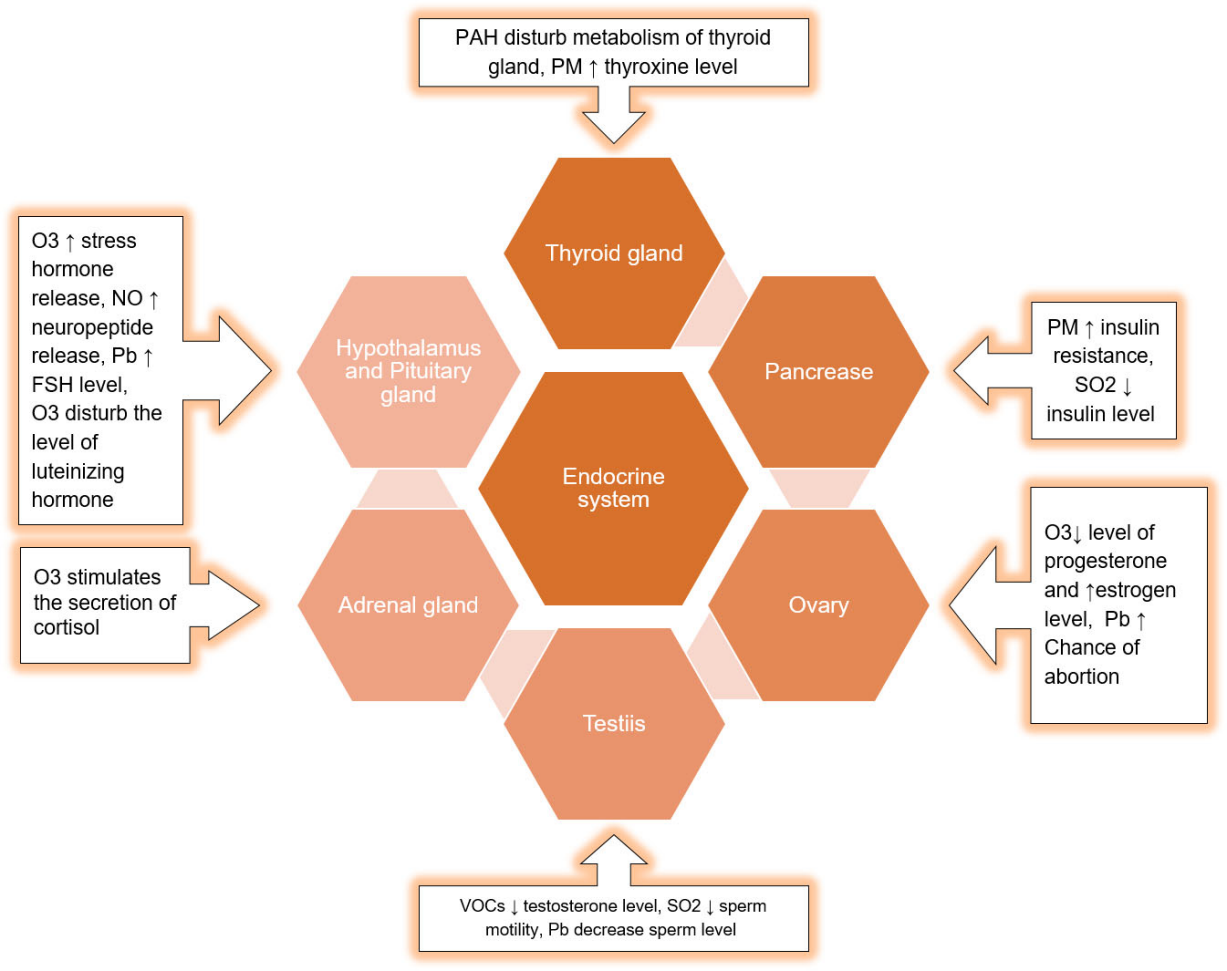
Impact of smog components on the different organs of the Endocrine system.

Exposure to phthalates and other toxicants also affects thyroid function and growth hormone homeostasis (129). This effect might be modulated by thyroid autoantibodies (130). The impact of smog components on the different organs of the endocrine system is described in **Figure 7**.

There is strong evidence that exposure to formaldehyde is associated with reproductive and developmental toxicities as it can cross the placental barrier. Menstrual irregularities and infertility were observed in females exposed to toxic level of formaldehyde (124). Some studies have demonstrated that a high cortisol level is significantly associated with exposure to smog pollutants. Cortisol plays a significant role in cognition and depression. The response starts in the brain and activates the hypothalamus-pituitary-adrenal axis to produce more cortisol which triggers inflammatory and apoptotic gene expression. This eventually leads to dementia and depression (131).

## 7 Cumulative endocrine disruptive effect of toxicants in smog

Several studies have documented the combined effect of smog toxicants on human endocrine system. These endocrine effects include an increase in stress hormones, infertility and insulin resistance.

### 7.1 Hormonal imbalance with combined toxicants in smog

It is evident from previous studies that a high concentration of NO_2_ and PM_2.5_ in the air was strongly linked to increased level of salivary cortisol level, CRH, and adrenocorticotropic hormone (ACTH) in adults. Moreover, the combined exposure to PM_2.5_ and NO_2_ was evaluated which showed an increased risk of cancer among women with a higher familial E_2_ polymorphism exposed only to NO_2_ only. It was also found that exposure to NO_2_, O_3_, and PM_2.5_ in young male individuals resulted in significant changes in estradiol levels (103). In addition, NO_2_, CO, SO_2_, or PM_2.5_ exposure exhibited a significant change in morning cortisol among children (94). PM_10_ exposure increased the risk of osteoporosis among women over 40 years of age when they were evaluated for exposure to NO_2_, CO, SO_2_, or PM_2.5_, and PM_10_. In addition, reduced testosterone, progesterone, and FSH were evident in middle-aged male individuals when exposed to PM_2.5_, NO_2_, and SO_2_ or CO (98).

### 7.2 Reproductive toxicity of combined toxicants in smog

Various animal experiments have displayed that long-term exposure to ambient air pollutants can result in the reduction of male fertility. It was found that the chronic exposure to PAHs and PM_2.5_ impaired sperm function and spermatogenesis (132). Exposure to SO_2_, NO, CO, and CO_2_ also decreased the sperm quality (132). Even at low concentration, NO_x_ may affect sperm motility and sperm morphology (133). Similarly, SO_2_ is a proven toxicant for the reproductive organs of mammals (134). Spermatogenesis may consequently improve with a reduction in the level of these toxic oxides (74, 135).

### 7.3 Insulin resistance with combined toxicants in smog

An elevated level of PM, SO_2_, and O_3_ may be associated with insulin resistance and metabolic alterations that lead to diabetes mellitus, obesity, and related health risks by triggering oxidative stress, endoplasmic reticulum stress, and activation of c-Jun N-terminal kinase signaling (136). The gaseous components of smog tend to cause more metabolic disorders than PMs (137).

## 8 Risk factors and preventive measures to reduce smog exposure

Factors responsible for the formation of smog are globalization, urbanization and heavy transport usage, elevation in temperature, sunny climate, bricks formation, and decline in forestation. There are numerous techniques and procedures to control smog formation. There is a need to convert the toxic volatile compounds emitted from factories into less toxic volatile compounds by changing the operating conditions and recycling the stream to decrease air pollution. Reduction in particle size also helps to mask the hazardous effects of smog. Particle size can be reduced by a gravity chamber, cyclone filtration, bag filters, and precipitate scrubbers (138).

Wet scrubbers should be used for absorption, adsorption, chemical oxidation, and bio-filtrations of gases and vapors discharged from chemical industries. Fossil fuel engines must be replaced with alternative engines. Hydrogen fuel additives are essential to diminish the discharge of pollutants and upsurge the combustion cycle. Photocatalytic treatment must be carried out to reduce particle size and nitrogen oxide pollution (139). Furnaces, condensers, carbon absorbers, scrubbers, and texture channels are additional devices that should be used for air pollution control. The main source of smog is open burning. There should be strict prohibitions and legislation on the open burning of rice stubble, solid waste, and other dangerous materials.

Environmental Protection Agencies (EPA) should issue policies to Air quality index departments to control air pollutants such as PM_2.5_. EPA should impose rules and regulations for oil refineries to produce Sulphur free oil to decline the level of PM and SO_2_ gas (140). A single tree fixes approximately 20 kg of CO_2_ annually. EPA should work with the forest department to intensify the growth of plants as plants are environmentally safe agents to fix carbon and other toxic elements thus declining smog. Large smoke industries should be shut down to reduce the discharge of pollution. The federal government should issue directions to all vehicle manufacturers for the installation of a catalytic converter in motor vehicles to prevent toxic vehicular emissions such as NO_x_, SO_x_ and CO_x_ (141).

## 9 Conclusion and perspectives

This review showed that the gaseous components and PM_2.5_ present in smog significantly increased the risk of endocrine toxicity through disruption of the HPA axis. These gases and PM can alter the expression of proinflammatory cytokines and metabolizing enzymes to exhibit insulin resistance and metabolic alterations. These chemicals alter the level of sex hormones to predispose infertility in males and females and can culminate in birth defects.

Smog, nowadays, has become a global issue with alarming human health risks. Smog and its constituents are associated with altered functioning of the ovary, testis or pituitary, adrenal, and thyroid glands. Moreover, diabetes mellitus, insulin resistance, infertility, reduced motility and DNA damage in sperms, reduced conception, and DNA methylation are the consequences of altered gene expression due to smog exposure. Preterm birth, preeclampsia, and small for gestational age are the adverse outcomes of exposure to smog air pollutants. Suitable steps should be taken to avoid smog exposure and related health issues. Avoiding wood and coal burning, reducing energy use, using renewable sources of electricity, using public transport, implementing strict limitations on industrial gaseous discharge, and staying inside during times of poor air quality can help in reducing toxic exposure to smog. Federal agencies should enforce environmental protection laws to achieve a smog-free atmosphere or attempts should be made to reduce the exposure duration, amount, and risk of damage to the population.

It is evident from the previous literature that PM_2.5_ and gaseous constituents result in neurodegeneration as well as psychiatric disorders such as depression and anxiety through altered HPA axis. Learning and memory deficits and psychomotor dysfunction are closely linked to endocrine disruption caused by smog. Therefore, we strongly suggest that further research should be guided to establish a link between PM_2.5_ and neuroendocrine, CNS toxicity, and hormonal disruption.

## Author contributions

AS: Conceptualization, Data curation, Supervision, Writing – original draft, Writing – review & editing. TA: Conceptualization, Data curation, Writing – original draft, Writing – review & editing. MA: Conceptualization, Data curation, Writing – original draft, Writing – review & editing.
